# Outcome and management of 2–59-month-old Nigerian children with chest indrawing pneumonia at primary-level healthcare facilities: a prospective cohort study

**DOI:** 10.7189/jogh.16.04004

**Published:** 2026-01-12

**Authors:** Adegoke G Falade, Obioma C Uchendu, Ayobami A Bakare, Tim Colbourn, Omotayo E Olojede, Joseph K Abuo, Olabisi Olasupo, Hamish R Graham, Rochelle A Burgess, Julius Salako, Ayodamola A Bakare, Funmilayo Shittu, Agnese Iuliano, Eric D McCollum, Yasir Bin Nisar, Shamim A Qazi, Carina King

**Affiliations:** 1 Department of Paediatrics, University of Ibadan, Ibadan, Oyo State, Nigeria; 2 Department of Paediatrics, University College Hospital, Ibadan, Oyo State, Nigeria; 3 Department of Community Medicine, University of Ibadan, Ibadan, Oyo State, Nigeria; 4 Department of Community Medicine, University College Hospital, Ibadan Nigeria; 5 Department of Global Public Health, Karolinska Institutet, Stockholm, Sweden; 6 Institute for Global Health, University College London, London, UK; 7 Department of Health Promotion and Education, University of Ibadan, Ibadan, Oyo State, Nigeria; 8 Department of Epidemiology and Medical Statistics, University of Ibadan, Ibadan, Oyo State, Nigeria; 9 Centre for International Child Health, University of Melbourne, Melbourne, Victoria, Australia; 10 School of Medicine, Johns Hopkins University, Baltimore, Maryland, USA; 11 Department of Sexual, Reproductive, Maternal, Child and Adolescent Heath and Ageing, Geneva, Switzerland; 12 Independent Consultant, Geneva, Switzerland

## Abstract

**Background:**

In 2012, World Health Organization (WHO) recommended outpatient oral amoxicillin for children aged 2–59 months with chest indrawing pneumonia without general danger signs, based on randomised trials. We assessed mortality and case management for such children routinely managed at primary healthcare centres (PHCs) in Lagos, Nigeria.

**Methods:**

This prospective observational cohort study (September 2021–September 2023) was conducted in Ikorodu Local Government Area (LGA), nested within the Integrated Sustainable childhood Pneumonia and Infectious disease Reduction in Nigeria (INSPIRING) Lagos study across 16 PHCs. PHC healthcare workers (HCWs) trained in Integrated Management of Childhood Illness (IMCI) provided routine care, while INSPIRING staff independently assessed eligibility using IMCI criteria. The primary outcome was the 14-day case fatality rate (CFR) among children with chest indrawing pneumonia without general danger signs; secondary outcomes included antibiotic use, treatment adherence, and referral practices.

**Results:**

PHC HCW identified 24 chest indrawing cases, while INSPIRING staff diagnosed 247 cases, including 19 of the 24 identified by PHC HCWs. Among those followed up (n = 16), the CFR was 6.3% (n/N = 1/16; 95% confidence interval (CI) = 0.2–30.2) for PHC HCW identified cases; with the same death identified by INSPIRING staff (n/N = 1/197; CFR = 0.5%). The single event in each cohort, and high loss to follow-up, imply that these CFR estimates are statistically fragile and should be interpreted as indicative only. Only 4% (n/N = 1/24) of children received routine care aligned with IMCI protocols. Of those prescribed antibiotics, 50% (n/N = 4/8) completed the full course, and just 1 of the 6 of referred children was admitted to hospital.

**Conclusions:**

PHC HCWs rarely diagnosed chest indrawing pneumonia, and one-third of the patients were lost to follow up leading to a smaller than expected sample and therefore an imprecise CFR. Improving HCW capacity to identify and manage pneumonia, alongside strengthening IMCI implementation, is critical to reducing preventable child deaths in this setting.

Community-acquired pneumonia remains one of the leading causes of death among Nigerian children under five, responsible for an estimated 169 000 deaths (20% of global under-five pneumonia deaths) in 2021 – the highest worldwide [1]. Beyond its acute burden, pneumonia causes long-term respiratory complications and impaired lung function [2]. Improving the management is a proven mortality-reduction strategy [3].

Most low- and middle-income countries (LMICs) implement the comprehensive WHO Integrated Management of Childhood Illness (IMCI) strategy – including pneumonia – for prompt assessment, classification, antibiotic therapy, supportive care, and referral [4–6]. Following multiple trials [7–9], World Health Organization (WHO)’s 2012 guideline recommended outpatient oral amoxicillin for chest indrawing pneumonia in children aged 2–59 months – previously treated as an inpatient condition [10]. This was incorporated into the 2014 IMCI chart booklet and reaffirmed in 2024 [6,11].

Despite this global recommendation, implementation has varied. A WHO survey conducted in 2018–2019 found that only 30% of countries had adopted the revised IMCI protocol to treat chest indrawing pneumonia with oral antibiotics at home, while 41% still recommended referral to higher-level facilities [12]. Among countries adopting home treatment, 65% reported using oral amoxicillin as the primary therapy for 2–59-month-old children with chest indrawing pneumonia [12]. The revised WHO guidelines offer multiple benefits – including a 39.5% reduction in treatment costs [13], and the potential to improve access to care, alleviate pressure on overstretched health systems, and promote responsible antibiotic use in high-burden LMIC settings.

However, concerns remain about the safety and effectiveness of outpatient management. A 2018 retrospective analysis in Kenya reported elevated mortality rate among children with chest indrawing, palmar pallor, or severe acute malnutrition [14], raising questions about the external validity of the evidence used to justify the guideline change [15,16]. A secondary analysis of hospital data from several studies in LMICs found that children with chest indrawing in clinical trials, where pulse oximetry was sometimes available, had a 75% lower risk of death compared to those in routine care [17]. Such findings highlight the importance of assessing outcomes outside of tightly controlled research environments.

Multiple studies have highlighted health worker diagnostic sensitivity issues, particularly the under-recognition of chest indrawing and hypoxaemia due to limited training, lack of clinical confidence, and absence of diagnostic tools like pulse oximeters [11–13]. Additionally, task-shifting to lower cadres, such as community health workers, has raised concerns about treatment fidelity, antibiotic misuse, and inadequate referral practices [14,15]. Ensuring continuous supervision, refresher training, and supportive policies remains a key barrier to effective decentralisation of pneumonia care.

Despite promising evidence from other LMICs [18], Nigeria has not systematically studied the outcomes of chest indrawing pneumonia treatment at the primary health centre (PHC) level under routine programme conditions. While a previous pilot project from one State in Nigeria explored community management [19], there remains no published prospective study evaluating mortality, treatment adherence, and health system outcomes for outpatient chest indrawing pneumonia care in Nigerian PHC facilities. We therefore aimed to estimate 14-day mortality among 2–59-month-old children diagnosed and managed for chest indrawing pneumonia at public PHC facilities in Lagos State, Nigeria, as part of the multi-country Chest Indrawing PneumoniA Management (CIPAM) study. Secondary objectives included characterising antibiotic prescribing patterns, treatment adherence, and subsequent referral to hospitals [20].

## METHODS

### Study design

We conducted a prospective observational cohort study in 16 PHCs in Ikorodu Local Government Area (LGA), Lagos State, Nigeria, from September 2021 to September 2023, nested within the Integrated Sustain­able childhood Pneumonia and Infectious disease Reduction in Nigeria (INSPIRING) Lagos project evaluating a PHC ‘stabilisation room’ intervention with dedicated INSPIRING staff trained in using IMCI pneumonia protocols [21]. Due to slow recruitment and concerns over potentially missing eligible cases, we added a retrospective cohort study, as a post-hoc sensitivity analysis. INSPIRING staff reviewed facility registers (January–June 2024) to identify records of children aged 2–59 months diagnosed with pneumonia between January and December 2023 in seven of the 16 study PHCs.

### Setting

**Figure 1:**
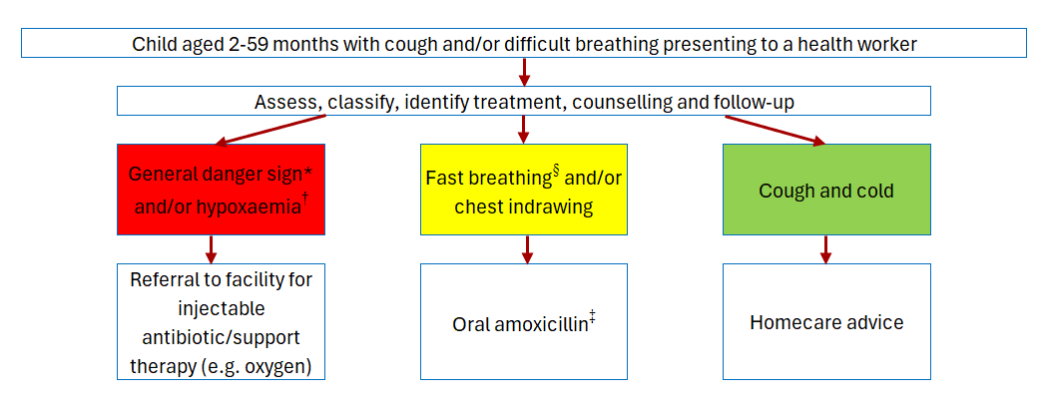
Integrated Management of Childhood Illness clinical pneumonia standard case management. *Unable to drink or feed, vomiting everything, convulsions, lethargic or unconscious, stridor in a calm child, or clinically severe malnutrition. †Oxygen saturation (Spo_2_)<90%, if pulse oximeters are available. SpO_2_ indicates peripheral arterial oxyhemoglobin saturation measurement by pulse oximetry. ‡The Paediatric Association of Nigeria's treatment recommendation for chest indrawing pneumonia also includes amoxicillin/clavulanic acid (co-amoxiclav), cefpodoxime, and cefuroxime as alternatives if amoxicillin is unavailable or the child is not responding. §Respiratory rate ≥50 in infants 2–11 months, and ≥40 in children 12–59 months.

Nigeria was selected for the multi-country CIPAM study given its high prevalence of childhood pneumonia and its adoption of the 2014 WHO IMCI guidelines for chest indrawing pneumonia management within PHCs (**Figure 1**) [6]. In Lagos State the under-five mortality rate was 46 per 1000 livebirths in the 2023–2024 Demographic and Health Survey [22], and an estimated 17 995 pneumonia cases in 2018 [23]. This study was conducted in Ikorodu LGA, a peri-urban setting, with an estimated population of 900 000 in 2017, with 28 government PHCs and two government secondary referral facilities.

We included 16 government PHCs – seven were 'flagship' PHCs providing 24-hour services with doctors on duty, and nine PHCs with high patient volumes, open 8 *am* – 4 *pm*, Monday to Friday. PHCs provide outpatient care but may stabilise and observe severe or emergency cases for 2–6 hours, pending referral or discharge. Community Health Extension Workers (CHEWs), nurses, and doctors (present in 'flagship' PHCs only) provide care. CHEWs are trained in schools of health technology for at least two years and are licensed and regulated by the Community Health Practitioners Registration Board of Nigeria.

Healthcare workers from all government facilities in the LGA took part in IMCI training, delivered by the Lagos State Ministry of Health, with financial support from Save the Children Nigeria. These trainings were done in batches throughout 2021–2022, with each lasting five days for IMCI – focused on the pneumonia classification algorithm, including recognition of chest indrawing. An additional 1-day was spent on pulse oximetry and oxygen treatment, delivered by the Oxygen for Life Initiative. Healthcare workers were provided with IMCI chart booklets and facilitation materials (*e.g*. pictorial guides), and a basic package of IMCI equipment (respiratory rate timers, Mid Upper Arm Circumference (MUAC) tapes, weighing scales, stadiometers, personal protective equipment). An additional refresher training was delivered in January 2023 by the research team. The seven 'flagship' PHCs were also provided with pulse oximeters and oxygen concentrators as part of the INSPIRING Lagos project [21]. Amoxicillin suspension was more commonly available than dispersible amoxicillin tablets at PHC facilities.

### Study population

Children aged 2–59 months presenting to the participating PHCs with WHO IMCI defined chest indrawing pneumonia according to routine healthcare worker (HCW) assessment were eligible (**Figure 1**). The main PHC HCW diagnosis is recorded as free text in facility-based case notes, with several terms used for 'pneumonia'. We, therefore, defined 'pneumonia' as any of the following: pneumonia, bronchopneumonia, acute lower respiratory tract infection or lower respiratory tract infection.

### Prospective cohort

For the prospective cohort, INSPIRING staff, who were nurses collected data in all facilities during regular working hours. They completed a 1-week residential IMCI pneumonia training in July 2020 at University College Hospital, Ibadan, covering theory and practical chest indrawing assessment. Refresher trainings occurred in January 2021, July 2021, and January 2023, with monthly supervisory visits by a site manager.

INSPIRING staff screened all children aged 2–59 months presenting with acute illness for WHO IMCI-defined pneumonia before PHC HCW assessment [21]. Children meeting IMCI criteria were enrolled after consent, and caregiver’s sociodemographic characteristics, vaccine history, and contact details were recorded. PHC HCWs then independently assessed, diagnosed, and prescribed treatment, documenting findings in outpatient registers and case notes, from which INSPIRING staff extracted data.

On day 15 post-enrolment, INSPIRING staff phoned caregivers to confirm child survival, onward care, and treatment adherence. Caregivers were called up to three times before being classified as lost to follow-up. For deaths, calls were stopped, and the site manager conducted a household verbal autopsy after mourning, using the WHO 2016 tool updated for COVID-19 [24].

### Retrospective cohort

As a sensitivity analysis for missed PHC HCW-diagnosed chest indrawing pneumonia, INSPIRING staff reviewed registers from seven facilities (1 January–31 December 2023) to identify children aged 2–59 months. Using the same IMCI screening form, they extracted clinical data, identified WHO IMCI chest indrawing cases, and phoned caregivers to confirm survival. For children who had died, verbal autopsies were not done, and deaths were attributed to the most recent presentation.

### Sample size

The descriptive analysis sample size was 292, based on estimating a single proportion with a 5% case fatality rate (CFR) for chest indrawing pneumonia, 3% margin of error, and 95% confidence level. Allowing for 5% loss to follow-up, we targeted enrolment of 310 children aged 2–59 months [20].

### Statistical analysis

Categorical variables were summarised as frequencies and percentages, and continuous variables as mean (±standard deviation (SD)) or median (interquartile range (IQR)). The primary outcome was the 14-day CFR following PHC HCW diagnosis of chest indrawing pneumonia. As some children re-presented, analyses used presentations as the denominator. INSPIRING staff diagnosed cases were also described and compared with PHC HCW cases.

We conducted three sensitivity analyses:

1) estimating the CFR within the retrospective cohort (Figure S1 in the **Online Supplementary Document**);

2) eligibility criteria also including PHC HCW diagnosed acute respiratory tract infection with chest indrawing (Table S1 in the **Online Supplementary Document**);

3) excluding hypoxaemia (Oxygen saturation (SpO_2_)<90%) as a danger sign (Table S1 in the **Online Supplementary Document**).

We also merged the prospective and retrospective cohorts to check for overlapping children between the two data sets, to explore routine facility data quality. All analyses were performed using STATA version 17 (StataCorp LLC, College Station, TX, USA).

## RESULTS

### Population description and comparative diagnosis

**Figure 2:**
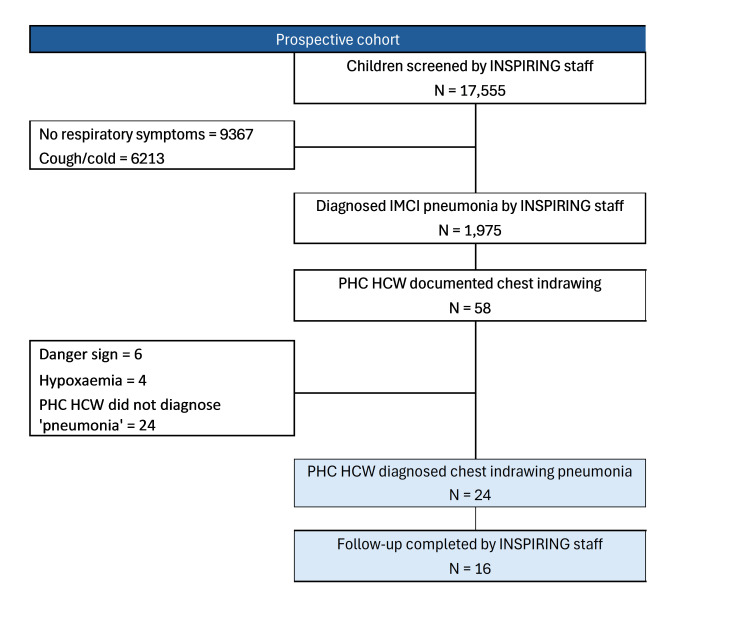
Participant recruitment and inclusion diagram for HCW diagnoses cases.

During the prospective cohort period, 17 555 children aged 2–59 months were screened by INSPIRING staff, of whom n/N = 8188/17 555 (47%) presented with cough and/or difficult breathing. Of these n/N = 1975/8188 (24%) were diagnosed with WHO-defined IMCI pneumonia and consented to follow-up visits. Of these 1975 recruited children, PHC HCWs routinely diagnosed chest indrawing pneumonia in n/N = 24/1975 (1.2%) (**Figure 2**). In contrast, the INSPIRING staff diagnosed n/N = 247/1975 (13%) of children with chest indrawing pneumonia (Figure S2 in the **Online Supplementary Document**). Of the 24 cases diagnosed by PHC HCWs as chest indrawing cases, 79% (n/N = 19/24) were also classified the same by INSPIRING staff. Of the five cases where there was disagreement, four were classified as severe pneumonia cases by INSPIRING staff, and one as a fast-breathing pneumonia case (**Table 1**). Among the INSPIRING staff chest indrawing pneumonia cases, n/N = 228/247 (92%) were missed by PHC HCWs – instead recorded as Acute Respiratory Infection (ARI)/cough, fast breathing pneumonia, malaria, or other conditions (**Table 1**). Follow-up completion was higher in the INSPIRING-diagnosed group compared to the PHC HCWs (80% *vs*. 67%).

**Table 1:** Comparison of PHC HCW and INSPIRING staff diagnosed cases of chest indrawing pneumonia

HCW diagnosis*	INSPIRING staff diagnosis (n = 247)		
Chest indrawing pneumonia, (n = 247)	Fast breathing pneumonia	Danger sign pneumonia
Chest indrawing pneumonia (n = 24)	19	1	4†
ARI/cough	82		
Fast breathing pneumonia	22		
Severe pneumonia	1		
Asthma	2		
Malaria	48		
Sepsis	37		
Other	15		
No diagnosis	68		

ARI – acute respiratory infection, HCW – healthcare workers, INSPIRING – Integrated Sustainable childhood Pneumonia and Infectious disease Reduction in Nigeria, PHC – primary healthcare centres

*Healthcare workers could assign more than 1 diagnosis

†The danger signs reported were hypoxaemia (n = 1), vomiting everything (n = 1), unable to feed or drink (n = 1), lethargic (n = 2).

Chest indrawing pneumonia was diagnosed by PHC HCWs in only six of 16 facilities, with five flagship facilities accounting for n/N = 22/24 (92%) cases. Information about facilities and IMCI training is presented in Table S2 in the **Online Supplementary Document**. INSPIRING staff diagnosed chest indrawing pneumonia in all 16 facilities, with n/N = 157/247 (64%) from flagship sites; overall, 54% (n/N = 1071/1975) of all IMCI pneumonia cases came from flagship facilities. INSPIRING staff diagnosis of chest indrawing pneumonia remained consistent over time and showed no clear association with training periods. (Table S3 in the **Online Supplementary Document**). Only two of the eight prospective and 14 retrospective cases that overlapped in time were matched between the cohorts (Figure S1 in the **Online Supplementary Document**).

### Clinical presentation of chest indrawing cases

Among the 1975 children assessed, PHC HCWs documented chest indrawing in 58 cases, but only 24 (41%) were classified as chest indrawing pneumonia. While 10 had danger signs or hypoxaemia; the rest were mostly misdiagnosed as acute respiratory infection (ARI)/cough (n = 11), malaria (n = 4), or had no recorded diagnosis (n = 7) (**Figure 2**).

More infants aged 2–11 months were diagnosed with chest indrawing by both PHC HCWs and INSPIRING staff (67 and 64%, respectively). However, INSPIRING staff diagnosed a higher proportion of girls with chest indrawing pneumonia than PHC HCWs (40 *vs*. 29%) (**Table 2**).

**Table 2:** Demographic and clinical characteristics of chest indrawing pneumonia cases: comparison between PHC HCW and INSPIRING staff diagnoses

Characteristics	PHC HCW diagnosed (n = 24)	INSPIRING staff diagnosed (n = 247)*
Age		
*2–11 mo*	16 (67%)	157 (64%)
*12–59 mo*	8 (33%)	90 (36%)
Sex		
*Female*	7 (29%)	99 (40%)
*Male*	17 (71%)	148 (60%)
Follow-up complete	16 (67%)	197 (80%)
14-day case fatality	1/16 (6.3%)	1/197 (0.5%)
HCW-recorded vital signs		
Respiratory rate (RR) recorded	8 (33%)	42 (17%)
*RR 2–11-mo-olds (mean, SD)*	56 (11.4)	51 (14.7)
*RR 12–59-mo-olds (mean, SD)*	61 (21.5)	47 (17.1)
Temperature recorded	19 (79%)	151 (61%)
*Temperature °C (mean, SD)*	37.3 (1.3)	37.2 (1.1)
SpO_2_ recorded	12 (50%)	34 (14%)
Weight recorded	6 (25%)	27 (11%)
Malaria status recorded	14 (58%)	106 (43%)

HCW – healthcare workers, INSPIRING – Integrated Sustainable childhood Pneumonia and Infectious disease Reduction in Nigeria, PHC – primary healthcare centres, SD – standard deviation, SpO_2_ – oxygen saturation

*For INSPIRING staff diagnosed cases, variables shown are those extracted from the routine case notes as documented by PHC HCWs.

During routine assessment, vital signs were generally poorly recorded by PHC HCWs, with 33% (n/N = 8/24) of children having a documented respiratory rate and 25% (n/N = 6/24) a recorded weight. This was even lower for the INSPIRING staff cases, where the PHC HCWs documented respiratory rate (RR) for 17% (n/N = 42/247) of children. For those with measurements, HCW-diagnosed cases had a higher mean RR according to the PHC HCW counts (56 *vs*. 51 for 2–11-month-olds; 61 *vs*. 47 for 12–59-month-olds), while mean temperatures were similar (37.3°C *vs*. 37.2°C) (**Table 2**).

SpO_2_ was documented by PHC HCWs for 50% (n/N = 12/24) of children that they diagnosed with chest indrawing pneumonia, compared to PHC HCWs documenting it for 14% (n/N = 34/247) of children which the INSPIRING staff had diagnosed. Malaria testing was more frequently recorded for PHC HCW-diagnosed cases (58 *vs*. 43%), with malaria positivity rates of 37 *vs*. 30%, respectively. Laboratory investigations, especially full blood count, were more often documented for HCW’s own cases (67 *vs*. 50%). The data on clinical signs and laboratory investigations were similar in the retrospective cohort (Table S1 in the **Online Supplementary Document****)**.

### HCW management and treatment adherence

**Figure 3:**
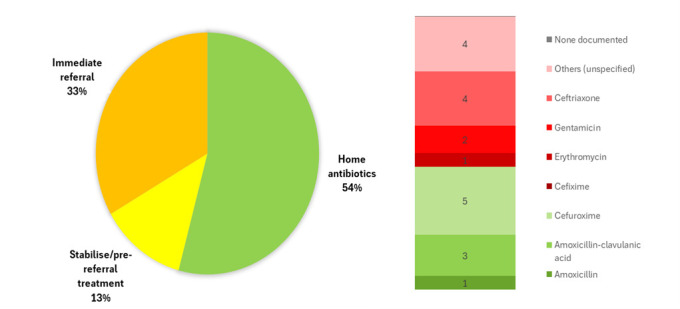
Healthcare worker treatment recommendations for chest indrawing pneumonia (n = 24). Green indicates treatment according to guidelines. Children could be prescribed more than one antibiotic (20 prescriptions are presented for 12 children).

Overall, n/N = 13/24 (54%) HCW-diagnosed chest indrawing cases were given home oral antibiotics, n/N = 3/24 (13%) were stabilised in the facilities, and n/N = 8/24 (33%) were immediately referred (**Figure 3**). Among the 16 with completed follow-up, 7 (44%) caregivers sought care elsewhere and two were hospitalised. Of those advised home antibiotics, 50% (n/N = 4/8) filled the prescription and completed the course (**Table 3**).

**Table 3:** Management and treatment adherence for chest indrawing pneumonia: PHC HCWs *vs*. INSPIRING staff (prospective cohort)

Management/outcome	PHC HCW diagnosed cases (n = 24)	INSPIRING staff diagnosed cases (n = 247)*
Treatment recommendation		
Home treatment (oral antibiotics)	13 (54%)	189 (77%)
Facility stabilisation	3 (13%)	15 (6%)
Immediate referral	8 (33%)	43 (17%)
Follow-up complete	16 (67%)	197 (80%)
Treatment adherence among home treatment cases with follow-up	n = 8	n = 148
*Antibiotic prescription filled*	6 (75%)	108 (73%)
*Full antibiotic course completed*	4 (50%)	96 (65%)
Treated per national IMCI protocol (oral amoxicillin)	1 (4%)	68 (28%)
Treated per PAN guideline†	9 (38%)	126 (51%)
Care seeking after index visit	7/16 (44%)	45/197 (23%)
Hospital admission after index visit	2/16 (13%)	8/197 (4%)

HCW – healthcare workers, IMCI – Integrated Management of Childhood Illness, PHC – primary healthcare centres, INSPIRING – Integrated Sustainable childhood Pneumonia and Infectious disease Reduction in Nigeria, PAN – Paediatric Association of Nigeria

*For INSPIRING staff-diagnosed cases, values are based on PHC HCW documentation in routine records.

†Recommended alternatives include oral amoxicillin-clavulanic acid, oral cefpodoxime, and oral cefuroxime.

Among the 13 children advised home treatment, one had no documented antibiotic, while the remaining 12 received 20 prescriptions (median (MD) = 1 per child; interquartile range (IQR) = 1–2). Common antibiotics were oral cefuroxime, injectable ceftriaxone, and oral amoxicillin-clavulanic acid. Only n/N = 1/24 (4%) received IMCI-recommended amoxicillin. In addition to oral amoxicillin, the Paediatric Association of Nigeria (PAN) treatment recommendation for chest indrawing pneumonia includes oral amoxicillin-clavulanic acid, oral cefpodoxime, and oral cefuroxime as alternatives if amoxicillin is unavailable or the child is not responding [25], with 38% (n/N = 9/24) of children treated according to this guideline. For the INSPIRING staff diagnosed cases, PHC HCWs treated 28% (n/N = 68/247) with oral amoxicillin according to IMCI protocol, 51% (126/247) according to the PAN guidelines, with 77% (n/N = 189/247) of children overall recommended for home treatment.

### Case fatality rate

Among the 16 PHC HCW-diagnosed children followed up, one death occurred (CFR = 6.3%; 95% confidence interval (CI) = 0.2–30.2). This was also the only death among INSPIRING-diagnosed cases (CFR = 0.5%; 95% CI = 0.01–2.8; n/N = 1/197). With single events, small denominators, and high loss to follow-up, these CFRs are fragile and should be viewed as indicative only, best interpreted in the context of poor case detection, documentation, and management quality rather than as definitive mortality estimates.

In the retrospective cohort, one death was also recorded, of the 10 children with completed follow-up (CFR = 10.0%; 95% CI = 0.3–44.5) (Figure S1 in the **Online Supplementary Document****)**. One death each in the prospective and retrospective cohorts occurred in females who were recommended outpatient antibiotic treatment (cefuroxime plus ceftriaxone; amoxicillin-clavulanic acid plus 'other') and did not have an SpO_2_ measurement done.

## DISCUSSION

We report survival among 2–59-month-old children with chest indrawing pneumonia managed by PHC HCWs in routine care settings in peri-urban Lagos, Nigeria. The prospective cohort showed a CFR of 6.3% among those followed up, though low recruitment and 33% loss to follow-up limited precision. However, the INSPIRING staff concurrently diagnosed considerably more cases, had lower loss to follow-up (20%), and a CFR of 0.5%, identifying the same single death as PHC HCWs. Overall, chest indrawing was rarely documented by PHC HCWs, reflecting poor IMCI implementation in diagnosis and treatment. Compliance with vital-sign assessment (respiratory rate, SpO_2_, weight) was low. Strengthening IMCI adherence is critical. Caregiver compliance was also poor, with only 50% completing antibiotic treatment.

Of the 24 chest indrawing cases diagnosed by PHC HCWs, 19 were also diagnosed by INSPIRING staff – indicating PHC healthcare workers had a high true positive rate, but also a high false negative rate. Given the INSPIRING staff diagnosed cases had fewer respiratory assessments done by the PHC HCWs, were more often treated as outpatients for a cough/cold and had lower mortality – it suggests PHC HCWs missed chest indrawing in children who presented with less obvious respiratory symptoms, but did routinely diagnose children who were more visibly sick with a respiratory infection.

The studies for outpatient management of chest indrawing pneumonia all reported mortality rates of less than 1% [26–28], in-line with the CFR reported by the INSPIRING staff. In our study, the INSPIRING staff more closely reflected trial conditions, as their role was to actively look for pneumonia cases, carefully inspect children for the presence of chest indrawing, and submit data according to a structured respiratory examination. Therefore, it is not surprising that they would identify more cases, including more subtle presentations of chest indrawing that may be missed by PHC HCWs who were busy. Our observed CFR for PHC HCW cases, however was similar to that reported in a recent systematic review, based on largely hospital-based observational studies (CFR = 9.1%) [29], and from a secondary analysis of over 150 000 pneumonia inpatients [17]. This discrepancy between real-world and trial contexts highlights the urgent need to understand why IMCI implementation in this setting was so poor, and improve paediatric pneumonia care quality.

A recent multi-country trial in four LMICs found community health worker home management of chest indrawing pneumonia safe and non-inferior to facility-based treatment [18], though hypoxaemic children were excluded – highlighting the importance of pulse oximetry for safe outpatient care. PHC HCWs performed pulse oximetry in 50% of chest indrawing cases, considerably higher than the 11% reported in the main INSPIRING study [30], but still suboptimal and below other settings [31,32]. This suggests HCWs valued SpO_2_ measurement when chest indrawing was recognised. Notably, both deaths in the prospective or retrospective cohort lacked documented SpO_2_.

The INSPIRING data collectors identified 12.5% of pneumonia cases as chest indrawing, eight times more than PHC HCWs’. This aligns with findings from Malawi, where 16.0% of PHC pneumonia cases were chest indrawing [33]. Most PHC HCW detections came from flagship facilities with doctors. A recent meta-analysis reported the sensitivity of non-physician healthcare workers in recognising chest indrawing was low at 46% (32–56%) [34] and a study from Malawi found that higher cadres of HCWs diagnosed pneumonia more often [35]. Though chest indrawing is a subjective clinical sign, poor detection here may reflect varied staff mix and limited IMCI training.

Recognising weak IMCI implementation, the study team added a retrospective cohort, which mirrored prospective findings but exposed poor record quality. Qualitative interviews with PHC HCWs from study facilities revealed issues with step-down training, high staff turnover, and lack of on-going supervision and support to embed knowledge into practices [36]. These mirror wider systemic challenges in pneumonia care. Respiratory rate assessments were rarely done; only 33% were recorded – better than other studies [37–39]. Low detection by PHC HCWs likely reflects systemic training deficits, limited professional development, and low motivation.

This highlights broader system barriers to IMCI implementation, with additional HCW training alone unlikely to improve care quality. Effective pneumonia management needs system-level investments such as routine supervision with IMCI audit checklists, clear task-shifting frameworks for lower-cadre HCWs, digital decision-support tools (*e.g*. mobile IMCI algorithms, SpO_2_ triggers), and facility-based mentorship, peer review and supportive supervision. There is urgent need to identify which of these strategies work best within peri-urban public health systems in Lagos and similar Nigerian settings.

Only 38% of children received treatment aligned with Paediatric Association of Nigeria guidelines [25]. Frequent antibiotic polypharmacy, even without danger signs or hypoxaemia, raises antimicrobial stewardship concerns. Stronger supervision and clearer prescribing protocols are needed to curb unnecessary use and potential resistance [40], especially given children were often initiated on second line treatments.

PHC HCW-identified clinical signs often misalign with IMCI and Paediatric Association of Nigeria pneumonia treatment guidelines, leading to overtreatment of mild illness like cough and cold, and undertreatment of severe cases. This underscores the need to harmonise and adapt these protocols for better local relevance and implementation.

The first step is to understand why quality IMCI implementation remains difficult despite active training which had limited impact [30]. Although conducted in relatively well-resourced PHCs in densely populated urban Lagos, these findings have broader relevance for similar urban and peri-urban LMIC settings. Many sub-Saharan African PHCs face similar challenges – limited diagnostics, reliance on syndromic management, and task-shifting to lower-cadre HCWs in frontline pneumonia care. Caution is needed, however, in generalising to rural or conflict-affected areas with weaker infrastructure and staffing.

Examining factors like training gaps, diagnostic uncertainty, caregiver expectations, and drug availability could explain the protocol deviations observed. However, qualitative data or direct observations of patient-provider interactions were beyond this study’s scope. Future mixed-methods research is needed to clarify adherence barriers and guide effective interventions.

Several factors could influence the internal and external validity of our findings. First, the study was under-powered to estimate the CFR with the desired precision of 5%. Children presenting outside of PHC operating hours were not enrolled in the study, as INSPIRING study staff were only present during day-time working hours. This may have missed sicker children, which PHC HCWs diagnosed as having chest indrawing pneumonia. The retrospective cohort explored this but also showed few PHC HCW diagnoses, suggesting it was not due to our recruitment methodology. Thus, a definitive CFR for routinely managed chest indrawing pneumonia cannot be stated. Second, poor documentation limited reliability of routine case notes in this setting – only two of eight prospective cases were matched, and 14 retrospective cases were not found, likely due to reliance on caregiver-reported cough and difficulty breathing to trigger a pneumonia assessment by our data collectors. Finally, higher loss to follow-up among PHC HCW cases further increased CFR uncertainty.

## CONCLUSIONS

Although the sample was insufficient to fully meet the study objective, this itself is instructive. The study provides real-world data on chest indrawing pneumonia management among 2–59-month-olds in peri-urban Lagos PHCs, revealing valuable insights into diagnostic and treatment practices under routine conditions (*i.e*. PHC HCW practice), where chest indrawing was rarely documented and vital signs were often not done. Improving the quality of pneumonia care at the PHC level in Nigeria will need a more nuanced appreciation for how care is delivered, HCWs are supported, and quality of care is monitored.

## Additional material


Online Supplementary Document
