# Supplementary material for: Outcome and management of 2–59-month-old Nigerian children with chest indrawing pneumonia at primary-level healthcare facilities: a prospective cohort study
Source: J Glob Health. 2026 Jan 12;16:04004. doi: 10.7189/jogh.16.04004 (PMC12793930; doi:10.7189/jogh.16.04004)
Supplement: Online Supplementary Document [file jogh-16-04004-s001.pdf]

**Supplement to: Falade AG, Uchendu OC, Bakare AA, Colbourn T, Olojede OE, Abuo JK, Olasupo O, Graham HR, Burgess RA, Salako J, Bakare AA, Shittu F, Iuliano A, McCollum ED, Nisar YB, Qazi SA, King C. Outcome and management of 2–59-month-old Nigerian children with chest indrawing pneumonia at primary-level healthcare facilities: prospective cohort study. J Glob Health. 2026;16:04004.**

**Figure 1: Retrospective cohort**

For the retrospective case note review, 7,608 case notes were reviewed, of which 2940 records had any respiratory symptoms documented, and 18/2940 (0.6%) children were routinely diagnosed with chest indrawing pneumonia by PHC healthcare workers (**Figure S1**). Follow-up was completed for 10/18 (56%) children.

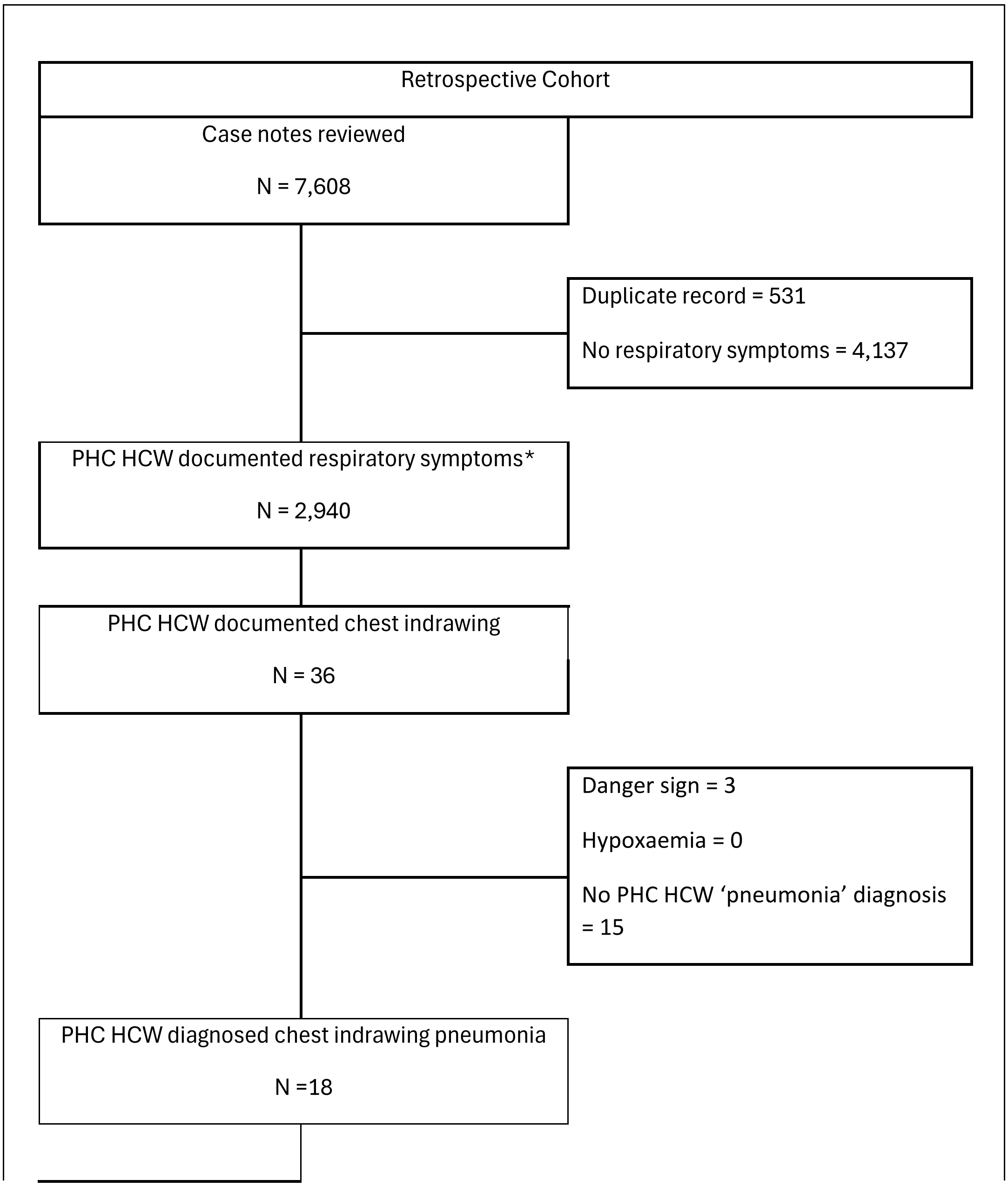

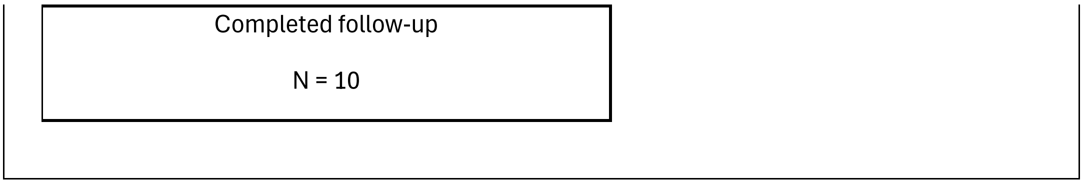


*Respiratory symptoms included: cough/cold, difficult breathing, fast breathing, and noisy breathing.

The clinical presentation of children with PHC HCW diagnosed chest indrawing pneumonia in the retrospective cohort was similar to the prospective cohort, although there were more girls in the retrospective cohort (29% versus 44%). The 14-day CFR was 10%, with one death recorded – **Table S4**. Amongst the 18 children, 13 (72%) were recommended home treatment, 1 (6%) was given no treatment, 1 (6%) was stabilised in the facility, and 3 (17%) were referred. Of the 13 recommended home treatment, 4 had no antibiotic prescription documented. The remaining 9 children had 12 antibiotics prescribed (mean 1.3/per child, SD = 0.5). Overall, 15% (n=2/18) of children were treated according to the 2014 WHO IMCI protocol and 54% (n=7/18) according to the Paediatric Association of Nigeria's treatment recommendations.

**Supplementary Figure 2: Participant recruitment and inclusion diagram for INSPIRING staff diagnosed cases**

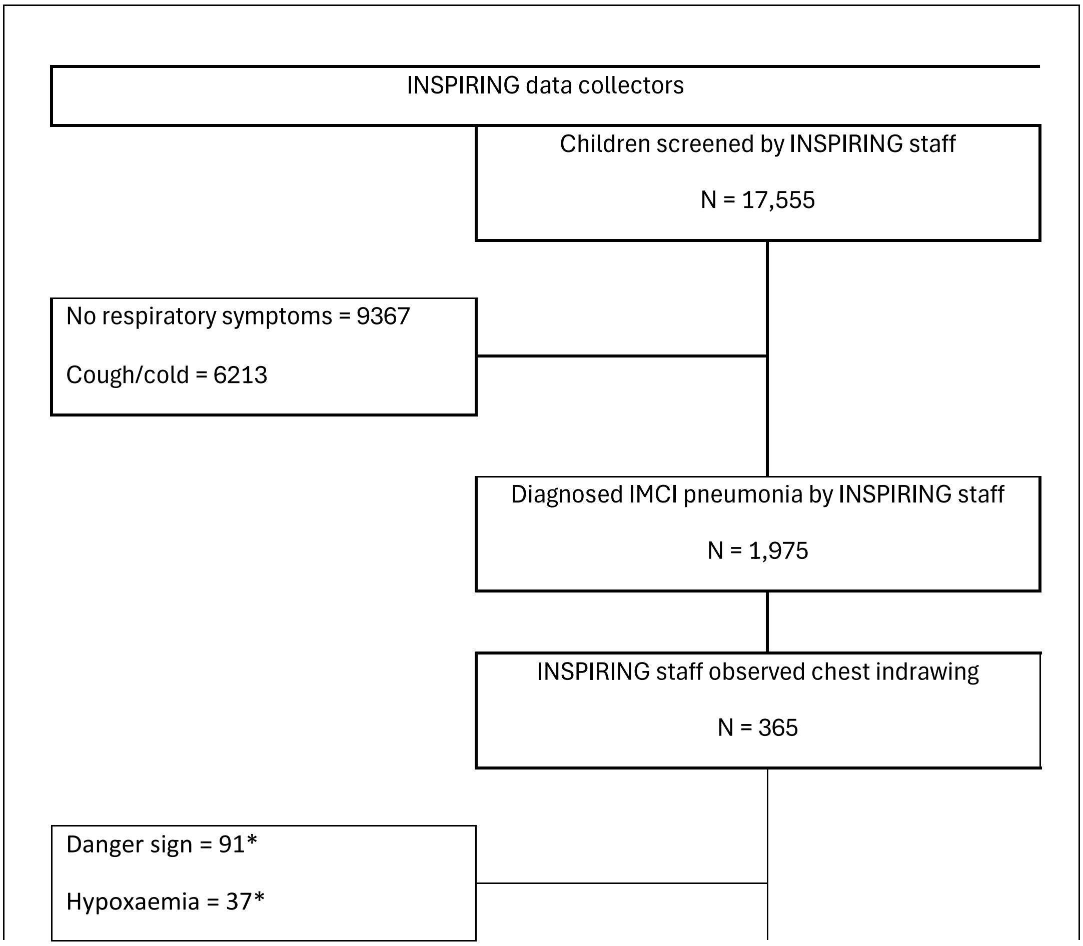

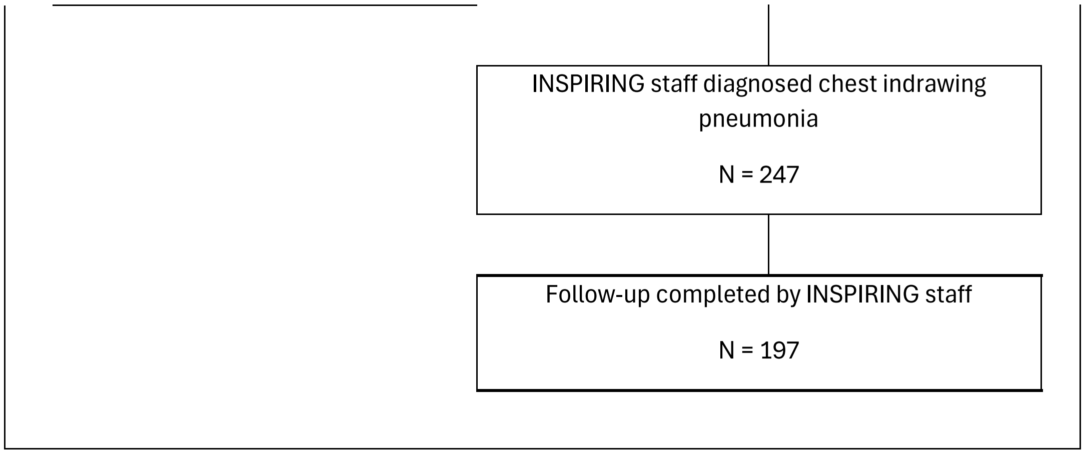


*10 children had both hypoxaemia and general danger signs, and therefore this adds up to 128 instead of 118

**Supplementary Table 1: Sensitivity analyses for the definition of chest indrawing pneumonia**

| | | PHC HCW diagnosed chest indrawing – including ARI (n=35) | PHC HCW diagnosed chest indrawing – ignoring $SpO_2$ (n=27) | INSPIRING staff diagnosed chest indrawing – ignoring $SpO_2$ (n=274) |
|---|---|---|---|---|
| Age | 2-11 months | 23/35 (66%) | 18/27 (67%) | 176/274 (64%) |
| | 12-59 months | 12/35 (34%) | 9/27 (33%) | 98/274 (36%) |
| Sex | Female | 11/35 (31%) | 9/27 (33%) | 110/274 (40%) |
| | Male | 24/35 (69%) | 18/27 (67%) | 164/274 (60%) |
| Respiratory rate* | Mean, SD | 53.6 (16.3) | 58.0 (17.0) | 52.6 (16.9) |
| | Normal RR | 4/35 (11%) | 4/27 (15%) | 23/274 (8%) |
| | Fast breathing | 8/35 (23%) | 7/27 (26%) | 31/274 (11%) |
| | Not recorded | 23/35 (66%) | 16/27 (59%) | 220/274 (80%) |
| Temperature† | Mean, SD | 37.1 (1.1) | 37.3 (1.2) | 37.2 (1.1) |
| | Normal | 18/35 (51%) | 12/27 (44%) | 105/274 (38%) |
| | Fever | 10/35 (29%) | 10/27 (37%) | 65/274 (24%) |
| | Not recorded | 7/35 (20%) | 5/27 (19%) | 104/274 (38%) |
| Oxygen saturation | MD, IQR | 97 (95 - 98) | 96 (93 - 97) | 95 (88 - 98) |
| | >=94% | 14/35 (40%) | 10/27 (37%) | 26/274 (9%) |
| | 90-93% | 3/35 (9%) | 2/27 (7%) | 6/274 (2%) |
| | <90% | | 3/27 (11%) | 11/274 (4%) |
| | Not recorded | 18/35 (51%) | 12/27 (44%) | 231/274 (84%) |
| Weight-for-age z-score | Mean, SD | -0.61 (1.91) | -0.77 (2.05) | -0.66 (2.13) |
| | ≥ -2.0 | 5/35 (14%) | 4/27 (15%) | 22/274 (8%) |
| | -3.0 to -2.0 | 2/35 (6%) | 2/27 (7%) | 5/274 (2%) |
| | Not recorded | 28/35 (80%) | 21/27 (78%) | 247/274 (90%) |
| Malaria status‡ | Positive | 6/35 (17%) | 5/27 (19%) | 34/274 (12%) |
| | Negative | 12/35 (34%) | 11/27 (41%) | 84/274 (31%) |
| | Not recorded / inconclusive | 17/35 (49%) | 11/27 (41%) | 156/274 (57%) |
| Laboratory tests requested | Chest x-ray | 1/35 (3%) | 1/27 (4%) | 4/274 (1%) |
| | Urine analysis | 1/35 (3%) | 1/27 (4%) | 1/274 (<1%) |
| | Full blood count | 20/35 (57%) | 17/27 (63%) | 135/274 (49%) |
| Follow-up | | 26/35 (74%) | 20/27 (74%) | 220/274 (80%) |
| Case fatality | | 1/26 (3.9%) | 2/20 (10.0%) | 3/220 (1.4%) |

* Fast breathing is defined as respiratory rate ≥50 in infants 2-11 months, and ≥40 in children 12-59 months

†Temperature was only documented in the prospective cohort dataset.

‡ Result according to either malaria rapid diagnostic test or microscopy

$SpO_2$ indicates peripheral arterial oxyhaemoglobin saturation measurement by pulse oximetry.

MD: Median; IQR: Interquartile range; SD: Standard deviation.

**Supplementary Table 2: Chest indrawing case recruitment and HCW training, by study facilities**

| Facility | Children diagnosed with pneumonia by INSPIRING staff | PHC HCW chest indrawing pneumonia cases | INSPIRING staff chest indrawing pneumonia cases | PHC HCWs with any IMCI training* | PHC HCWs with IMCI training in the prior 2 years* |
|---|---|---|---|---|---|
| 1 | 344 | 7 (2%) | 49 (14%) | 8/9 (89%) | 6/9 (67%) |
| 2 | 22 | 0 | 2 (9%) | 0/4 (0%) | 0/4 (0%) |
| 3 | 384 | 1 (<1%) | 36 (9%) | 7/8 (88%) | 6/8 (75%) |
| 4 | 138 | 6 (4%) | 35 (25%) | 6/12 (50%) | 5/12 (42%) |
| 5 | 72 | 2 (3%) | 7 (10%) | 8/9 (89%) | 5/9 (56%) |
| 6 | 39 | 0 | 4 (10%) | 10/12 (83%) | 6/12 (50%) |
| 7 | 72 | 6 (8%) | 24 (33%) | 8/12 (67%) | 6/12 (50%) |
| 8 | 24 | 0 | 4 (17%) | 3/4 (75%) | 0/4 (0%) |
| 9 | 27 | 0 | 1 (4%) | | |
| 10 | 235 | 0 | 4 (2%) | 3/3 (100%) | 3/3 (100%) |
| 11 | 46 | 0 | 3 (7%) | 2/2 (100%) | 2/2 (100%) |
| 12 | 120 | 0 | 5 (4%) | 4/4 (100%) | ¾ (75%) |
| 13 | 203 | 0 | 34 (17%) | 3/5 (60%) | 2/5 (40%) |
| 14 | 159 | 2 (1%) | 24 (15%) | 0/6 (0%) | 0/6 (0%) |
| 15 | 24 | 0 | 12 (50%) | 2/2 (100%) | 2/2 (100%) |
| 16 | 66 | 0 | 3 (5%) | 3/3 (100%) | 2/3 (67%) |
| **Overall** | **1975** | **24 (1.2%)** | **247 (12.5%)** | **67/95 (71%)** | **48/95 (51%)** |

Grey indicates flagship PHCs, which are staffed by doctors.

*These data were collected as part of the main INSPIRING Lagos study process evaluation in November – December 2022. Facility 9 did not provide any data for this survey. This survey was done before additional training was provided by study staff in January 2023. Full details of the methodology are presented in Olojede et al (2024) [37].

**Supplementary Table 3: Chest indrawing recruitment before and after IMCI trainings**

a) INSPIRING Staff diagnoses: trainings presented are INSPIRING Study Staff trainings conducted by the Oxygen for Life Imitative (the population is children aged 2-59 months presenting with an acute illness)

| INSPIRING staff diagnoses | Training & piloting (July - August 2020) | **1st September – 31st December 2020** | Re-training 1 (January 2021) | **1st February – 30th June 2021** | Re-training 2 + 1st training WHO data collectors (July – August 2021) | **1st September 2021 – 31st December 2022** | Re-training 3 (January & February 2023) | **1st March – 30th September 2023** |
|---|---|---|---|---|---|---|---|---|
| No pneumonia | | 1202 (84.5%) | | 2141 (87.0%) | | 13936 (88.8%) | | 3331 (90.9%) |
| Fast breathing pneumonia | | 65 (4.6%) | | 79 (3.2%) | | 1211 (7.7%) | | 224 (6.1%) |
| Chest indrawing pneumonia | | 26 (1.8%) | | 35 (1.4%) | | 186 (1.2%) | | 51 (1.4%) |
| Severe pneumonia | | 130 (9.1%) | | 206 (8.4%) | | 368 (2.3%) | | 57 (1.6%) |

a) PHC HCW diagnoses: trainings presented are for PHC HCWs conducted by the Nigerian Ministry of Health, and the refresher by the Oxygen for Life Initiative (the population is children aged 2-59 months diagnosed with IMCI pneumonia by INSPIRING study staff)

| Diagnosis | | Ministry of Health IMCI residential trainings (March– August 2021)* | **1st September 2021 – 31st December 2022** | Refresher training conducted by Oxygen for Life Initiative (January & February | **1st March – 30th September 2023** |
|---|---|---|---|---|---|
| No pneumonia | | | 1470 (84.5%) | | 157 (88.2%) |
| Fast breathing pneumonia | | | 237 (13.6%) | | 17 (9.6%) |
| Chest indrawing pneumonia | | | 15 (0.9%) | | 4 (2.2%) |
| Severe pneumonia | | | 17 (1.0%) | | 0 |

*Overall, 6 batches of training were done, 4 between March – August 2021, one in April 2022 and one in September 2022.

**Supplementary Table 4: Description of demographic and clinical presentation of chest indrawing pneumonia cases routinely diagnosed in the retrospective cohort**

| | | n/N (%) |
|---|---|---|
| Age | 2-11 months<br>12-59 months | 12/18 (67%)<br>6/18 (33%) |
| Sex | Female<br>Male | 8/18 (44%)<br>10/18 (56%) |
| Respiratory rate* | Mean, SD | 59.4 (12.4) |
| | Normal RR<br>Fast breathing<br>Not recorded | 7/18 (39%)<br>0/18 (0%)<br>11/18 (61%) |
| Temperature† | Mean, SD | |
| | Normal<br>Fever (>=37.5°C)<br>Not recorded | |
| $SpO_2$ | Median (IQR) | 96 (94-98) |
| | >=94%<br>90-93%<br>Not recorded | 3/18 (17%)<br>1/18 (6%)<br>14/18 (78%) |
| Weight-for-age z-score | Mean (SD) | -1.68 (2.40) |
| | ≥-2.0<br>-3.0 to -2.0<br>Not recorded | 3/18 (17%)<br>1/18 (6%)<br>14/18 (78%) |
| Malaria status‡ | Positive<br>Negative<br>Not recorded / inconclusive | 1/18 (6%)<br>7/18 (39%)<br>10/18 (56%) |
| Laboratory tests requested | Chest x-ray | 0/18 (0%) |
| | Urine analysis | 0/18 (0%) |
| | Full blood count | 13/18 (72%) |
| Follow-up complete | | 10/18 (56%) |
| 14-day case fatality rate | | 1/10 (10%) |

* Fast breathing defined as respiratory rate ≥50 in infants 2-11 months, and ≥40 in children 12-59 months

†Temperature was only documented in the prospective cohort dataset.

‡ Result according to either malaria rapid diagnostic test or microscopy

$SpO_2$ indicates peripheral arterial oxyhaemoglobin saturation measurement by pulse oximetry.

**Supplementary Table 5: Description of children aged 2-59 months with PHC HCW diagnosed chest indrawing pneumonia, according to healthcare worker recommended referral**

| | | No referral (N=16) | Immediate referral (N=8) | p-value^ |
|---|---|---|---|---|
| Age | 2-11 months | 10 (63%) | 6 (75%) | 0.667 |
| | 12-59 months | 6 (38%) | 2 (25%) | |
| Sex | Female | 5 (31%) | 2 (25%) | 0.572 |
| | Male | 11 (69%) | 6 (75%) | |
| Fast breathing* | Normal RR | 2 (13%) | 1 (13%) | 0.822 |
| | Fast breathing | 4 (25%) | 1 (13%) | |
| | Not recorded | 10 (63%) | 6 (75%) | |
| Temperature† | Normal | 9 (56%) | 1 (13%) | 0.108 |
| | Fever | 5 (31%) | 4 (50%) | |
| | Not recorded | 2 (13%) | 3 (38%) | |
| $SpO_2$ | >=94% | 9 (56%) | 1 (13%) | 0.102 |
| | 90-93% | 1 (6%) | 1 (13%) | |
| | Not recorded | 6 (38%) | 6 (75%) | |
| Weight-for-age z-score | ≥ -2.0 | 4 (25%) | 0 (0%) | 0.247 |
| | -3.0 to -2.0 | 2 (13%) | 0 (0%) | |
| | Not recorded | 10 (63%) | 8 (100%) | |
| Malaria status‡ | Positive | 3 (19%) | 2 (25%) | 0.214 |
| | Negative | 8 (50%) | 1 (13%) | |
| | Not recorded / inconclusive | 5 (31%) | 5 (63%) | |
| Case fatality | | 1 (9.1%) | 0 | - |

^Fishers exact test

* Fast breathing is defined as respiratory rate ≥50 in infants 2-11 months, and ≥40 in children 12-59 months

†Temperature was only documented in the prospective cohort dataset.

‡ Result according to either malaria rapid diagnostic test or microscopy

$SpO_2$ indicates peripheral arterial oxyhaemoglobin saturation measurement by pulse oximetry.
